# Can We Predict Burnout among Student Nurses? An Exploration of the ICWR-1 Model of Individual Psychological Resilience

**DOI:** 10.3389/fpsyg.2016.01072

**Published:** 2016-07-19

**Authors:** Clare S. Rees, Brody Heritage, Rebecca Osseiran-Moisson, Diane Chamberlain, Lynette Cusack, Judith Anderson, Victoria Terry, Cath Rogers, David Hemsworth, Wendy Cross, Desley G. Hegney

**Affiliations:** ^1^School of Psychology and Speech Pathology, Curtin UniversityPerth, WA, Australia; ^2^School of Psychology and Exercise Science, Murdoch UniversityPerth, WA, Australia; ^3^School of Nursing, Curtin UniversityPerth, WA, Australia; ^4^School of Nursing and Midwifery, Flinders UniversityAdelaide, SA, Australia; ^5^School of Nursing, The University of AdelaideAdelaide, SA, Australia; ^6^School of Nursing, Midwifery and Indigenous Health, Charles Sturt UniversityBathurst, NSW, Australia; ^7^School of Nursing and Midwifery, University of Southern QueenslandBrisbane, QLD, Australia; ^8^Faculty of Applied and Professional Studies - School of Business, Nipissing UniversityToronto, ON, Canada; ^9^School of Nursing and Midwifery, Monash UniversityMelbourne, VIC, Australia

**Keywords:** resilience, students, nursing, burnout

## Abstract

The nature of nursing work is demanding and can be stressful. Previous studies have shown a high rate of burnout among employed nurses. Recently, efforts have been made to understand the role of resilience in determining the psychological adjustment of employed nurses. A theoretical model of resilience was proposed recently that includes several constructs identified in the literature related to resilience and to psychological functioning. As nursing students are the future of the nursing workforce it is important to advance our understanding of the determinants of resilience in this population. Student nurses who had completed their final practicum were invited to participate in an online survey measuring the key constructs of the ICWR-1 model. 422 students from across Australia and Canada completed the survey between July 2014 and July 2015. As well as several key demographics, trait negative affect, mindfulness, self-efficacy, coping, resilience, and burnout were measured. We used structural equation modeling and found support for the major pathways of the model; namely that resilience had a significant influence on the relationship between mindfulness, self-efficacy and coping, and psychological adjustment (burnout scores). Furthermore, as predicted, Neuroticism moderated the relationship between coping and burnout. Results are discussed in terms of potential approaches to supporting nursing students who may be at risk of burnout.

## Introduction

Internationally there is concern about a rising nursing workforce shortage that can be attributed to both recruitment and retention issues (Doiron et al., 2008; Drury et al., 2009; Eley et al., 2010; Francis and Mills, 2011; Health Workforce Australia, 2012). There is an urgent need for more research examining the psychological well-being of nurses as it plays a fundamental role in recruitment and retention and has a direct impact on patient safety (Mealer et al., 2012a). It is well-known that the demanding nature of nursing work means that nurses are exposed to both acute and chronic stressors which can lead to conditions such as depression, anxiety, secondary traumatic stress and burnout (STS) (Figley, 1995a,b; Conrad and Kellar-Guenther, 2006; Craig and Sprang, 2009; Hooper et al., 2010; Stamm, 2010; Hegney et al., 2013, 2014a; Drury et al., 2014). Nurses who exhibit changes to their psychological well-being are more likely to resign earlier from the nursing workforce, or may reduce their employment fraction, which has an economic cost to employers (Mealer et al., 2012a). There is wide acknowledgment that the imminent retirement of older nurses, alongside high student attrition, low registered nurse graduate employment, and low retention of early career registered nurses, will have a significant impact on the available nursing workforce (CIfHI, 2000; Australian Institute of Health Welfare, 2013, 2015; Hegney et al., 2014b).

The transition of nursing students into the workforce as registered nurses remains a concern today. As identified (Chang and Daly, 2012), research data continues to show that the transition process is very stressful for new graduates and a number of newly graduated nurses continue to be lost from the profession due to the stressful process of change. This loss of potential nurses into the profession makes it very important to assist nursing students to not only be able to cope with the challenges of their education and clinical placements, but also to make them ready for the transition to the workplace. Nursing students experience many significant challenges as they enter not only into a higher education facility, with many coming straight from well-supported school environments, but also entry into complicated healthcare environments (Chang and Daly, 2012). High levels of student stress have been linked to participation in clinical placements (Milosevic et al., 2012) as well as the pressure of academic coursework (Pulido-Martos et al., 2012). Some studies also revealed that nursing students were exposed to considerable bullying and harassment during clinical placements (Timm, 2014; Levett-Jones et al., 2015).

It has been noted (Andrew et al., 2015) that nursing students tend to have “non-traditional” student profiles in the sense that there are high numbers from lower socio-economic backgrounds, “first-in-family” students (i.e., first person in their family to attend university), mature age students and female students. They suggest that this profile makes it more likely that they will have external commitments that compete with their studies, such as work commitments and caring for dependents. Indeed, a review of the literature (Andrew et al., 2015) confirmed that nursing students identified the major stressors facing them were time demands of family and financial concerns. As such, it is highly probable that they may experience stress, fatigue and burnout before they even complete their final exams and enter into the profession as a registered nurse. How students manage the competing demands and stressors associated with academic work and clinical placements may serve as an indicator of their later ability or resilience to manage the stressors associated with employment as a nurse.

Psychological resilience has been described as the ability of a person to overcome adversity and adjust in a positive manner to maintain their well-being (Cope et al., 2015; Reyes et al., 2015) and has been linked to persistence in the nursing workforce as well as in the educational context (Hodges et al., 2010; Gray, 2012; Knight et al., 2012; Manzano García and Ayala Calvo, 2012; Cope et al., 2015). In a study of acute care nurses, Hodges et al. (2010) postulated that resilience may vary due to the context or setting in which nurses are practicing. They identified three core processes of resilience: verifying fit, where personal passion for an area or setting is noted, stage setting, where resources are located and support is found, and optimizing the environment, where the person adapts to their setting in order to optimize their ability to cope. McGarry et al. (2013) also considered the context of the setting in which nurse's work as an important factor influencing resilience. Their work in pediatric health care indicated that a younger workforce (particularly those below 25 years of age) was particularly susceptible to reduced well-being, including stress, depression, and anxiety. Other studies have indicated that higher rates of post-traumatic stress disorder exist in nurses working in intensive care, although some staff in those areas have been identified as being highly resilient and more likely to have optimism, cognitive flexibility, a personal moral compass, altruism, an ability to face fear, coping skills, a supportive network, exercise, and a sense of humor (Mealer et al., 2012a,b, 2014). Overall, there are similarities of what resilience actually involves in all of these studies: providing support to overcome stress, increased positivity, and improved flexibility (Waugh and Koster, 2015). Resilience is also seen as a way forward for professional groups (related to contextual settings) to sustain their specialty e.g., mental health (Cleary et al., 2014).

**Figure 1:**
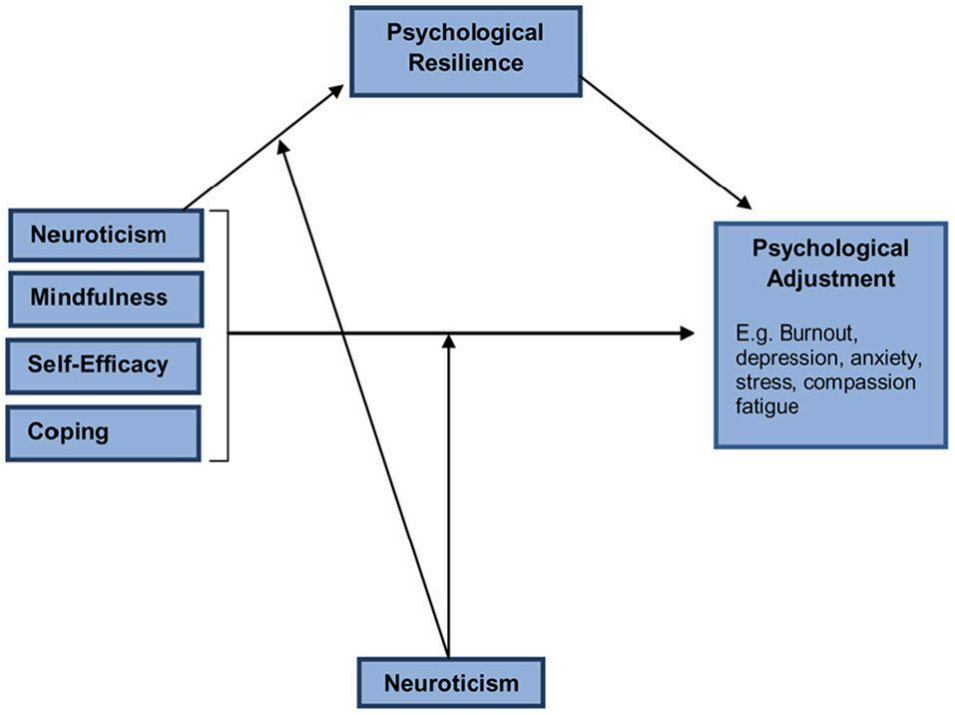
**The ICWR-1 model of individual psychological resilience**.

Recently, Rees et al. (2015) proposed a theoretically and empirically derived model of workforce resilience based on large-scale, multi-site surveys of the nursing workforce (Hegney et al., 2013, 2014a,b; Drury et al., 2014). This model includes several key constructs that are predicted to have significant relationships with psychological adjustment via the mediating influence of resilience. As shown in Figure 1, Neuroticism (also known as trait negative affectivity) is proposed as both a mediator of the relationship between mindfulness, self-efficacy, coping, and psychological adjustment but also as a moderator of the relationship between these variables and resilience. Neuroticism or trait negative affect is known to be a relatively stable temperamental factor associated with various negative psychological outcomes such as depression, anxiety, stress, burnout, and compassion fatigue (Drury et al., 2014; Lu et al., 2014; Sarubin et al., 2015; Craigie et al., 2016).

Other variables in the model include mindfulness, self-efficacy and coping. Mindfulness refers to the ability to de-center and respond less reactively and more flexibly to events (Teasdale, 1999) and is a strong predictor of burnout (Viladarga et al., 2011). Self-efficacy refers to an individual's belief that they can effectively perform a task (Bandura, 1977) and is associated with psychological adjustment including levels of anxiety and resilience (Saks, 1994). Similarly, the use of adaptive coping strategies is associated with better overall psychological adjustment (Chang et al., 2007).

The aim of this study is to test the newly developed ICWR-1 model of individual psychological resilience among a group of student nurses. The link between resilience and psychological symptoms in student nurses has never been explored in any large-scale study. By understanding the key variables that influence student nurse resilience, strategies can be designed to specifically target these factors.

## Methods

### Participants

#### Inclusion criteria

Student nurses enrolled in a program leading to a bachelor of nursing qualification; student nurses in the final year of their program and having just undertaken their final year clinical placement. Students enrolled at the following universities in Australia: Monash University, Victoria; Flinders University, South Australia; The University of Adelaide, South Australia; Curtin University of Technology, Western Australia; The University of Southern Queensland, Queensland; and Charles Sturt University, New South Wales and in one Canadian University: Nippissing University.

#### Exclusion criteria

Student nurses enrolled in other programs not leading to a Bachelor of Nursing qualification.

### Measures

#### Demographic questionnaire

Age, gender, marital status, living arrangements, citizenship, country of birth, enrolment pattern, mode of study, scholarship support for program, current employment and where employed, family responsibilities impacting on studies and future working life, last clinical placement, working pattern on clinical placement, expectations of graduate program.

#### The connor-davidson resilience scale

Connor and Davidson (2003) is a survey based measure of stress, coping, ability, or resilience. Evidence from previous studies in the community (Hegney et al., 2008) of nursing populations (Gillespie, 2007) suggests that this scale is a valid and reliable measure of resilience for a range of normal and clinical populations (Connor et al., 2003). The original 25 item scale uses a five-point response scale with higher scores reflect greater resilience. Factor analyses have indicated that the 25-item measure is multifactorial, consisting of factors such as hardiness (10 items) social support/purpose (4 items), faith (2 items), and persistence (7 items). For the present study we elected to use the shorter 10-item version because factor analyses has found this version to be a pure measure of the central core construct of resilience that retains the excellent psychometrics of the longer version (Campbell-Sills and Stein, 2007). We considered this to be important for the present study where several other conceptually similar constructs are being investigated, such as self-efficacy and mindfulness. The use of the 10-item measure helped guard against the possibility of criterion-predictor contamination impacting on results. The observed alpha for the current study was excellent (0.90).

#### The professional quality of life scale version 5 (ProQol5) (Stamm, 2010)

The PROQOL5 utilizes 30, five point Likert scale items to measure each of three subscale components (Compassion Satisfaction; Secondary Traumatic Stress; Burnout, 10 items each). Although the PROQOL was originally developed for emergency personnel and trauma counselors, the scale has been utilized internationally and also has been psychometrically validated in different studies for various target populations (Stamm, 2010). For this study we examined scores on the Burnout Scale. The observed alpha for Burnout for the current study was good (0.75).

#### General self-efficacy scale (GSE)

Schwarzer and Jerusalem (1995) General self-efficacy refers to a broad and stable sense of personal competence to deal effectively with a variety of stressful situations. GSE is a universal construct, which means that it characterizes a basic belief that is inherent in all individuals. The GSE scale includes 10 items. A typical item is, “Thanks to my resourcefulness, I can handle unforeseen situations.” Possible responses are not at all true (1), hardly true (2), moderately true (3), and exactly true (4), yielding a total score between 10 and 40. High reliability, stability, and construct validity of the GSE has been confirmed and the scale has been adapted to 28 languages (Schwarzer and Jerusalem, 1995). For this study the observed alpha was very good (0.89).

#### Cognitive and affective mindfulness scale, revised (CAMS-R)

Feldman et al. (2007) is a 20 item self-report measure of trait-based mindfulness. Mindfulness items represent factors of awareness of internal experience, present moment focus, attention control, and acceptance of experience (e.g., I am easily distracted; I can accept things I cannot change; I can describe how I feel in the moment). Participants are asked to rate their responses to each item on a Likert scale with the following options: 1 (Rarely/Not at all), 2 (Sometimes), 3 (Often), or 4 (Almost always). Higher scores represent higher levels of mindful awareness. The authors have shown that the CAMS-R has acceptable internal consistency and evidence of convergent and discriminant validity with concurrent measures of mindfulness, distress, well-being, emotion-regulation, and problem-solving approaches. The measure has been subsequently evaluated in a clinical setting, which demonstrated that increases in mindfulness after cognitive therapy for depression were related to reductions in non-adaptive emotion regulation strategies and depression (Kumar et al., 2008). The observed alpha for the current study was good (0.80).

#### Brief cope

Carver (1997) is a 28-item self-report measure of both adaptive and maladaptive coping skills. The Brief COPE was developed based on concepts of coping from Lazarus and Folkman (1984). The Brief COPE is the abridged version of the COPE inventory and presents fourteen scales all assessing different coping dimensions: (1) active coping, (2) planning, (3) using instrumental support, (4) using emotional support, (5) venting, (6) behavioral disengagement, (7) self-distraction, (8) self-blame, (9) positive reframing, (10) humor, (11) denial, (12) acceptance, (13) religion, and 1(4) substance use. The observed Cronbach's alphas were above 0.70 (0.86, 0.76, 0.76, 0.89, 0.73, 0.82, 0.71, 0.78, 0.73, 0.72, 0.70, 0.83, 0.72 for adaptive, maladaptive, active coping, substance use, use of emotional support, use of instrumental support, behavioral disengagement, positive reframing, planning, humor, acceptance, religion and self-blame, respectively). The alphas for self-distraction (0.54), denial (0.62), and venting (0.58) were below (0.70).

#### The positive and negative affect scale (PANAS)

Watson et al. (1988) is a 20-item measure of an individual's overall affective state (e.g., afraid, jittery, active, alert, enthusiastic etc.). It consists of two separate 10-item sub-scales; one measuring positive affectivity (PA) and the other measuring negative affectivity (NA). Respondents are asked to rate the extent they have experienced a particular emotion using a five-point Likert Scale ranging from “very slightly or not at all” through to “very much.” A number of time frames can be used such as “over the past week” or “generally (on average).” In this study the time frame of “generally” was used to measure trait-based affectivity: TPA and TNA. The PANAS has demonstrated excellent reliability and validity, and is frequently used in research and clinical settings (Watson and Clark, 1984; Watson et al., 1988). The observed Cronbach's alphas were 0.88 positive affect and 0.85 for negative affect.

### Procedure

The study received approval from the Human Research and Ethics Committees at the seven universities. In total, between the two rounds of data collection in 2014 and 2015, the seven universities contacted 2970 students to complete the self-report questionnaire battery. Each university, sent out the reminders to the participants, thus the research team were unaware of the names of individuals. All responses were anonymous. The invitations sent out by the universities contained a letter of invitation, the Participant Information Sheet and a link to the Qualtrics hosting site. With the exception of the USQ in Australia where the data collection was mainly undertaken using the hard copy version of the Qualtrics survey. In Canada, paper-based surveys were handed out to nursing students in class (not the researcher's class). Students were asked to return the surveys in the accompanying sealed envelope to the School of Nursing administrative office or place them in a box at the front of the classroom when completed. For universities who used on-line Qualtrics as data collection, a total of two or three email reminders were sent to participants from each university over a period of six weeks that the study remained live on the website between 2014 and 2015. A total of 535 students responded to the study questionnaires (18.01% overall response rate). After data screening and missing data procedures were applied, a final sample of 422 students with valid data were included in the study. During assumption testing *n* = 7 participants responses were excluded as outliers. Data analysis was therefore conducted on a final sample of 415 students.

### Research design and analysis

The research was conducted as a cross-sectional, quantitative design at each data collection site. The endogenous variables in the ICRW model (see Figure 1) were resilience and psychological adjustment (i.e., burnout). The model's exogenous variables were mindfulness, adaptive coping style, maladaptive coping style, self-efficacy, and neuroticism. In addition to functioning as an exogenous variable, neuroticism was a moderating term of the indirect *a* model paths stemming from resilience to psychological adjustment for mindfulness, adaptive/maladaptive coping, and self-efficacy. Neuroticism was a moderating term of the direct *c'* model path between the aforementioned predictors and psychological adjustment. The adequacy of the model and its path coefficients in Figure 1 was examined via path analysis as elaborated on in the forthcoming Results. Follow-up probing of significant moderation terms was conducted via simple slopes analysis and the Johnson-Neyman technique as outlined in the forthcoming Results.

## Results

### Participant characteristics

Generally (see Table 1) the nursing students were female (89.10%), in their late twenties (28.33, *SD* = 10.09) and were citizens (88.63%) of the country where they studied. Students were mainly single (63.03%) with less than half having (42.35%), dependent family responsibilities affecting their capacity to work. Almost all students (82.42%) studied on campus and full time (88.15%). More than half (69.86%) expected to be accepted into a graduate program and few of them (13.78%) were recipients of a scholarship or an award. During their last placement, students were in tertiary (53.10%) or primary (21.43%) level of care. They had essentially morning or/and evening shift (49.05%) and day shift (28.44%). In terms of employment, more than half of students (68.41%) worked in parallel of their nursing studies and mainly in health environment (68.75%). In mean, they worked 21.26 (SD = 13.06) hours.

**Table 1:** **Demographic characteristics of the entire sample (***N*** = 415)**.

**Main Demographic Description**	**% (*n*)**
Female	89.10 (376)
Mean age in years (SD)/range	28.33 (10.09)/19-62
Single, divorce, widow, separated	63.03 (266)
Citizen of the country	88.63 (374)
Live with family (parents or spouse)	67.30 (284)
Dependent family responsibility affecting the capacity to work	42.35 (166)
Study on campus	82.42 (347)
Full time study	88.15 (372)
Be accepted into a graduate program	69.86 (292)
Scholarship or Award	13.78 (58)
Shift during last practicum	
Morning and evening shifts only	49.05 (207)
Day shifts (between 6 a.m. and 6 p.m.)	28.44 (120)
Place Last Practicum	
Tertiary care^*^	53.10 (223)
Primary care^*^	21.43 (90)
Been Employed last 4 weeks	68.41 (288)
*Health Environment*	68.75 (198)
*Mean hours working (SD)–range*	21.26 (13.09)/2-88

* *Tertiary care refers to specialist care, usually on referral from a primary or secondary health professional (e.g., tertiary hospital)*;

* *Primary care refers to health professionals who act as a first point of consultation for all patients*.

### Model testing

In preparation for model testing via Structural Equation Modeling (SEM), the ratio of estimated free parameters to cases suggested that path analysis would be most appropriate as an exploratory means of testing the moderated multiple mediator model, instead of an underpowered full structural regression model (Kline, 2010). Kline recommends a minimum case to free parameter ratio of 10:1 for sufficient power when conducting SEM, with >20:1 being ideal. Therefore our ratio of ~2:1 cases to parameters, before the inclusion of the moderation indicators and latent factors, suggested that a full structural regression model would be underpowered. The path analysis case to free parameter ratio of ~19:1 for the saturated model suggested that this approach was tenable.

Missing data from the 422 responses was calculated as being missing completely at random, Little's MCAR test χ^2^ (632) = 691.096, *p* = 0.051, therefore expectation maximization was used to address the points of missing data from the item responses. Total scores per participant for each measure were summed. Assumptions were examined prior to analysis, and suggested that univariate normality was problematic for neuroticism, maladaptive coping, and burnout; algebraic transformation was correctively applied. Influential multivariate and univariate outliers identified via Mahalanobis' distances, standardized residuals, and residual scatterplots were removed (*N* = 7), bringing the sample for analysis to *N* = 415. Moderating terms between neuroticism and each exogenous predictor were centered via their residuals per the method suggested by Little et al. (2007), to ensure orthogonality of the moderating terms with their product components. Ill-scaled variances for variables were correct via algebraic transformation, and all remaining assumptions were met. Table 2 presents bivariate correlations and variances for variables employed in the path analysis.

**Table 2:** **Bivariate correlations and variances of path analysis variables (***N*** = 415)**.

	**Resil**	**Burnout**	**Mind**	**Adpt C**	**Neuro**	**Maldpt C**	**SelfE**	Neuro^*^Mind	Neuro^*^Maldpt	Neuro^*^SelfE	Neuro^*^Adpt
**RESILIENCE**											
Burnout	−0.486^**^										
Mindfulness	0.627^**^	−0.481^**^									
Adaptive Coping	0.131^**^	0.027	−0.063								
Neuroticism	−0.336^**^	0.374^**^	−0.409^**^	0.239^**^							
Maladaptive Coping	−0.296^**^	0.509^**^	−0.435^**^	0.498^**^	0.477^**^						
Self-Efficacy	0.666^**^	−0.395^**^	0.557^**^	0.077	−0.324^**^	−0.224^**^					
Neuro^*^Mind	0.074	−0.126^*^	0.000	0.063	0.000	−0.121^*^	0.042				
Neuro^*^Adapt	−0.167^**^	0.068	−0.106^*^	−0.113^*^	0.000	0.000	−0.099^*^	−0.449^**^			
Neuro^*^SelfEff	0.027	−0.134^**^	0.008	0.027	0.000	−0.095	0.000	0.650^**^	−0.294^**^		
Neuro^*^Ada	0.062	−0.074	0.037	0.000	0.000	−0.019	0.031	−0.078	0.497^**^	0.029	
Variance	38.038	32.177	26.663	97.39	55.158	94.601	59.896	66.03	58.103	41.594	57.201

* *P ≤ 0.05*;

* P ≤ 0.01

**Figure 2:**
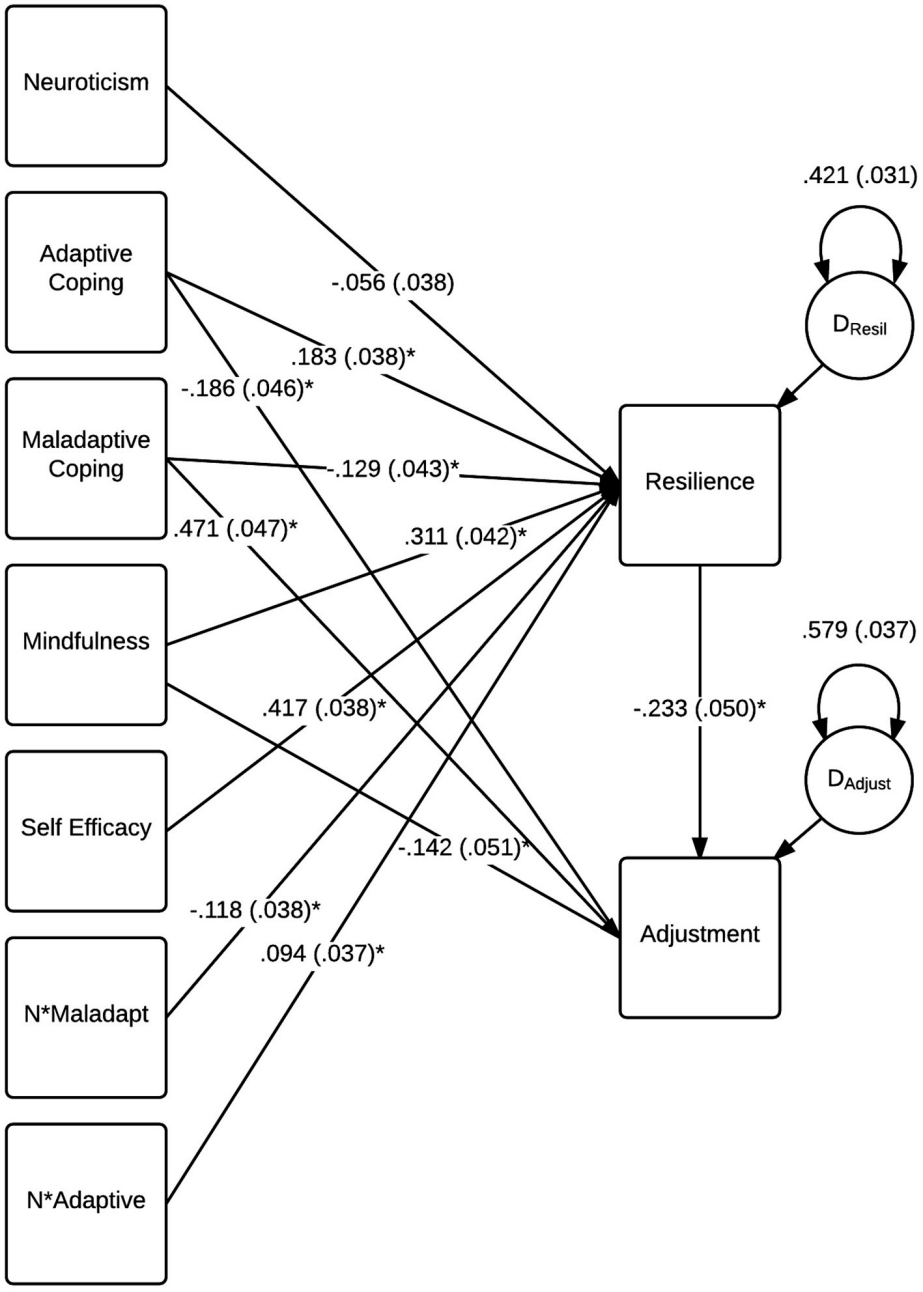
**Statistically significant standardized parameter coefficients for the model testing conditional direct and indirect effects on burnout (***N*** = 415)**. Standard errors are reported in brackets for each parameter.

Path analysis model testing was analyzed using maximum likelihood estimation, using the software mPlus version 7.31 (Muthén and Muthén, 2012). Resilience was regressed on neuroticism, mindfulness, self-efficacy, adaptive and maladaptive coping, and the four moderating terms. Burnout was regressed on all of the previous variables. Examination of the indirect and direct effects within the saturated model are described in the forthcoming sections. As the saturated model is just-identified (*df* = 0), we tested a variant of the saturated model that aimed to include only significant free parameters to estimate the adequacy of model fit. While neuroticism did not have a significant direct effect on either resilience or burnout as described shortly, it was a component of the moderator variables that influenced resilience scores and was therefore retained for model estimation (Hayes, 2013). The reduced free parameter model indicated a good fit to the data, $\chi_{\left(4\right)}^{2}$ = 7.085, *p* = 0.131, *RMSEA* = 0.043 (90% *CI* 0.000 to 0.094), *CFI* = 0.995, *TLI* = 0.980, *SRMR* = 0.011, and is presented in Figure 2.

#### Direct effects on burnout

Burnout had ~43.2% of its variance accounted for by its entered predictors. Table 3 provides the standardized and unstandardized predictors and their standard errors for this component of the model, based on estimates from the saturated model. Participants' reported level of mindfulness and the use of adaptive coping strategies were significant negative predictors of burnout assuming resilience remains constant. The use of maladaptive coping strategies was a significant positive predictor of burnout, indicating that participants who used these strategies more often had a poorer adjustment, assuming resilience remains constant. Maladaptive coping strategies had the largest direct effect on burnout (standardized *c'*_*Mal*_ = 0.435). Self-efficacy did not significantly predict burnout when holding the mediating term constant. No statistically significant conditional effects of neuroticism were observed for the latter effects, therefore evidence of its role as a moderator of the direct effects was not supported by these analyses.

**Table 3:** **Unstandardized and standardized estimates with standard errors for estimated model parameters (***N*** = 415)**.

	**Estimate**	***SE***	**Std. Estimate**	***SE***
**RESILIENCE**				
Neuroticism	−0.046	0.032	−0.055	0.038
Mindfulness	0.367	0.051	0.308^***^	0.042
Self Efficacy	0.331	0.031	0.416^***^	0.038
Adaptive Coping	0.116	0.024	0.185^***^	0.039
Maladaptive Coping	−0.086	0.028	−0.136^**^	0.044
Neuro^*^Mind	−0.001	0.035	−0.001	0.046
Neuro^*^Maladaptive Coping	−0.108	0.035	−0.133^**^	0.043
Neuro^*^Self-Efficacy	−0.033	0.040	−0.035	0.042
Neuro^*^Adaptive Coping	0.083	0.031	0.102^**^	0.038
**BURNOUT**				
Resilience	−0.168	0.053	−0.183^**^	0.057
Neuroticism	0.060	0.034	0.079	0.044
Mindfulness	−0.128	0.058	−0.116^*^	0.053
Self Efficacy	−0.051	0.038	−0.070	0.051
Adaptive Coping	−0.106	0.026	−0.185^***^	0.046
Maladaptive Coping	0.254	0.030	0.435^***^	0.050
Neuro^*^Mind	0.003	0.037	0.004	0.053
Neuro^*^Maladaptive Coping	−0.003	0.038	−0.005	0.050
Neuro^*^Self-Efficacy	−0.074	0.043	−0.084	0.049
Neuro^*^Adaptive Coping	−0.032	0.033	−0.043	0.045
**INTERCEPTS**				
Resilience	−1.336	3.152	−0.217	0.509
Burnout	50.384	3.373	8.882	0.660
**RESIDUAL VARIANCES**				
Resilience	15.963	1.108	0.420^***^	0.031
Burnout	18.273	1.269	0.568^***^	0.037

* *P ≤ 0.05*;

** *P ≤ 0.01*;

*** P ≤ 0.001

#### Indirect and conditional indirect effects on burnout

The indirect effects of adaptive and maladaptive coping, mindfulness, and self-efficacy, via resilience, were examined for an influence on participant burnout via calculated *ab* coefficients. The *ab* coefficient for each indirect effect is the product of the *a* path from a predictor (e.g., mindfulness) to resilience, and the coefficient of the *b* path from resilience to burnout, forming an *ab* coefficient whose magnitude can be assessed for statistical significance (Hayes, 2013). Examination of the indirect effect of mindfulness on burnout via resilience supported the significance of this effect, standardized *ab* = −0.056, *p* = 0.003. Consistent with the direct effect of mindfulness on burnout, the indirect effect of mindfulness on burnout via resilience was negative, suggesting that participants with higher scores on the mindfulness measure had lower burnout scores due to mindfulness' effect on resilience, which in turn influenced burnout. The indirect effect of self-efficacy on burnout via resilience was also significant, standardized *ab*_._ = −0.076, *p* = 0.002, and demonstrated the same direction of the indirect effect as mindfulness. Neither of these indirect effects was conditional on neuroticism influencing the *a* pathway between the predictor and resilience, therefore support for this component of the model was not observed in this analysis.

Both *ab* paths for adaptive and maladaptive coping indirectly influencing burnout via resilience were significant, standardized *ab* = −0.034, *p* = 0.007, and standardized *ab*_._ = 0.025, *p* = 0.026, respectively. The direction of these indirect effects were consistent with the direct effects on burnout, with the use of adaptive coping strategies associated with lower burnout via its effect on resilience, and the opposite pattern observed for the use of maladaptive coping strategies. However, the *a* pathways between adaptive/maladaptive coping and resilience were both conditional on participants' neuroticism scores, as evidenced by the significant moderating terms for these variables (*p* = 0.007 and *p* = 0.002, respectively, see Table 3). To further probe the nature of this moderating influence on the *a* pathway for the two coping predictors, the PROCESS macro (Hayes, 2013) for SPSS (version 22; IBM Corporation, Armonk, NY, USA) was used to perform simple slopes analyses and derive Johnson-Neyman significance regions to establish the properties of the moderating effects. Each simple slopes figure was calculated to reflect the relationship between either coping predictor and resilience at the 10th, 25th, 50th, 75th, and 90th percentiles of reported neuroticism scores for participants. This provided a means of examining the conditionality of the effect for participants with very low, low, average, high, and very high levels of neuroticism relative to the sample, respectively (Hayes, 2013). The regions of significance provided by the Johnson-Neyman Technique allowed us to examine whether the influence of neuroticism was significant across all levels of the moderator, or whether this moderating effect was influential only within a range of neuroticism scores (Hayes, 2013). Together, these supplementary analyses allowed greater clarity toward understanding the manner in which neuroticism was influential on the adaptive/maladaptive coping model pathways to resilience.

**Figure 3:**
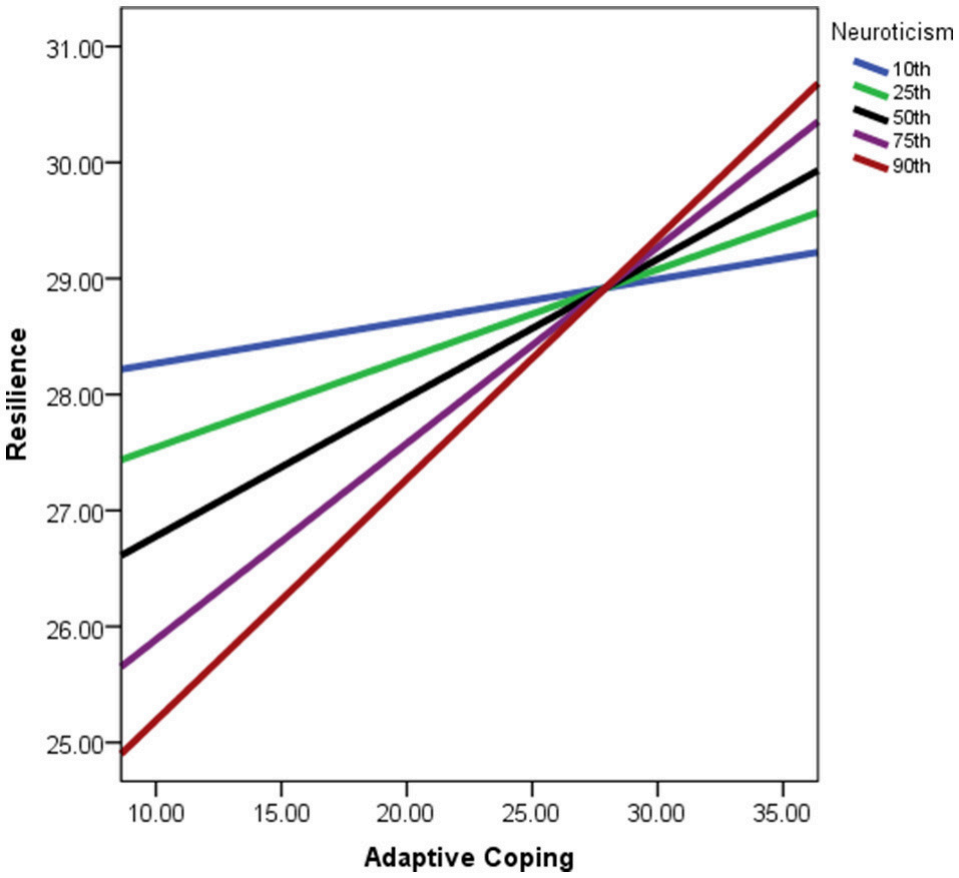
**Simple slopes graph of the relationship between adaptive coping and resilience being conditional on neuroticism scores**. Lines represent strength of relationship per percentiles of neuroticism scores within the sample. Adaptive coping scores reflect values post-algebraic-transformation.

As reported levels of neuroticism increased for participants, the relationship between adaptive coping being positively related to participant resiliency strengthened as demonstrated in Figure 3. Consequently, the strength of the indirect effect of coping being negatively related to burnout via resilience would be higher for participants with greater reported levels of neuroticism. This effect was not influential across all levels of neuroticism however; individuals with reported neuroticism scores approximately below the 19th percentile of the sample were not affected by this conditional effect. Therefore the conditionality of adaptive coping influencing resilience due to neuroticism was only apparent for participants with low to very high levels of neuroticism.

**Figure 4:**
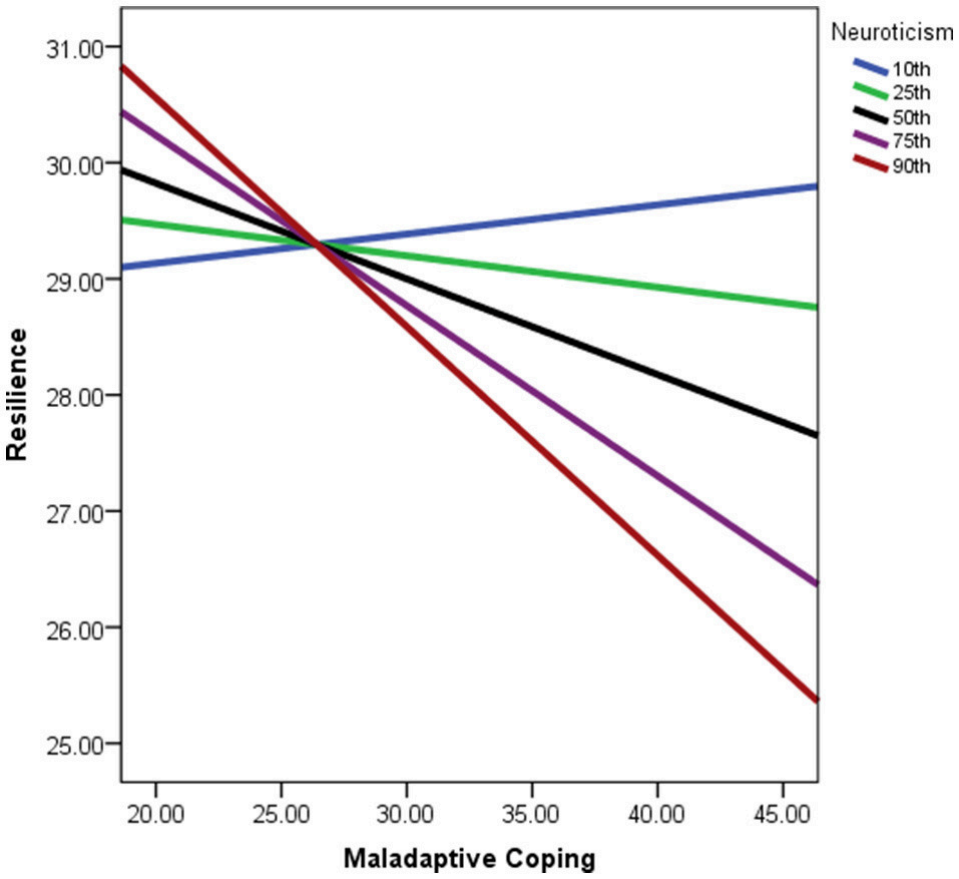
**Simple slopes graph of the relationship between maladaptive coping and resilience being conditional on neuroticism scores**. Lines represent strength of relationship per percentiles of neuroticism scores within the sample. Maladaptive coping scores reflect values post-algebraic-transformation.

In a similar manner to that of adaptive coping, the negative effect of maladaptive coping on reported resilience strengthened as neuroticism scores increased for participants (see Figure 4). The indirect effect of the use of maladaptive coping approaches being positively related to reported burnout via resilience was therefore stronger for participants with higher levels of neuroticism. The conditionality of this effect was limited to participants with moderate to higher levels of neuroticism in comparison to the rest of the sample though, as the region of significance for this effect extended only to participants above approximately the 42nd percentile for neuroticism scores. These findings regarding the conditionality of the indirect effects on burnout were therefore supported in the instances of adaptive and maladaptive coping behaviors, although these effects were not present for mindfulness and self-efficacy.

## Discussion

Student nurses are the future of the nursing workforce and thus efforts to understand how best to support their emotional well-being in relation to their work are of paramount importance. The aim of this study was to explore the applicability of a recently proposed model of workplace resilience (Rees et al., 2015) to explain the psychological functioning of student nurses. As predicted by the model, both mindfulness and coping had direct effects on burnout. Consistent with previous literature (Chang et al., 2007) and with the model, adaptive coping was associated with lower burnout and conversely maladaptive coping was associated with higher burnout. Similarly, the finding that higher levels of mindfulness were associated with lower levels of burnout is also consistent with previous literature (Viladarga et al., 2011) and with the model. Contrary to the predictions of the model, Neuroticism (Trait Negative Affect) did not moderate these direct relationships. Unlike coping and mindfulness, self-efficacy did not have a direct effect on burnout in this sample. However as explained below, self-efficacy had an indirect effect on burnout via resilience.

As predicted by the model, resilience had a significant influence on the relationship between mindfulness, self-efficacy and coping, and psychological adjustment (burnout scores). Higher mindfulness, higher self-efficacy, and coping scores were associated with lower burnout due to each variables effect on resilience. For mindfulness and self-efficacy, these effects were not moderated by Neuroticism, suggesting that the relationships between variables occur irrespective of a persons score on this personality variable. These findings offer some support for the development of programs for students that teach mindfulness skills, adaptive coping skills and strategies directly designed to bolster self-efficacy as a potentially important approach to strengthening student nurse resilience and thereby potentially preventing burnout.

Similar indirect effects were observed for both adaptive and maladaptive coping and burnout with these relationships also due to the effect of coping on resilience. However, for the coping variables, this relationship was moderated by Neuroticism. Specifically, as level of Neuroticism increases, the positive effect of adaptive coping on resilience strengthens. Similarly, as Neuroticism increases the negative effect of maladaptive coping on reported resilience strengthens. In other words, the indirect effect observed between coping and burnout (which occurs via the effect of coping on resilience) is greater for individuals with higher reported levels of Neuroticism. This finding is consistent with the predictions in the model and with previous research that strongly links Neuroticism with psychological vulnerability (Drury et al., 2014; Lu et al., 2014; Sarubin et al., 2015; Craigie et al., 2016).

This result suggests that compared to their counterparts who may score lower on Neuroticism, students who are high in Neuroticism would benefit the most from educational programs that emphasize the use of adaptive coping strategies such as planning, positive reframing, and seeking support. Such programs are likely to bolster the resiliency of these students. Such programs could be regarded as preventative in the sense that vulnerable students, high on neuroticism could be targeted and provided with these strategies early in the course of their studies. Importantly, these results also suggest that students high in Neuroticism may find activities like substance use, behavioral disengagement, and self-blame far more damaging to their resiliency when dealing with the stress of study and work, in comparison to their student counterparts who are lower in Neuroticism.

The current sample of nursing students were predominantly female and 42% reported having dependent family affecting their capacity to work. A large proportion (68%) were also working whilst studying. As noted Andrew et al. (2015) these external demands add pressure to an already demanding degree. The results of this study indicate that several variables are key in understanding the resilience and associated psychological adjustment of student nurses. Self-efficacy, coping, and mindfulness all impact on the overall resilience of students. Courses that aim to build perceptions of self-efficacy, teach the skills of mindfulness, and adaptive coping are likely to assist student nurses to remain resilient during their studies. Such skills would then have transferability into the employed nurse working environment and potentially help to buffer against the effects of stress. This study also identified that there may be a specific group of students high in Neuroticism and who also have poor coping skills, who are particularly at-risk and would be the cohort that would most benefit from such courses in self-care. It would be possible to screen for such students and offer them short courses aimed at building adaptive coping skills that could have the potential to prevent later burnout.

There are some limitations of this study that should be taken into consideration. First, we did not have sufficient power to run a fully saturated SEM model. As such, it is possible that some of the observed findings may change when a fully saturated model is tested. Second, the majority of students in this study were Australian. In order to gain an understanding of the universal applicability of the model it is necessary to test it with a broader international sample. Members of our team are currently undertaking such work with a large sample of students from Singapore and Hong Kong.

Theoretical models of workforce resilience are critical for understanding and predicting the factors impacting on nurse well-being and the related outcomes of retention and turnover. This is the first study to test a theoretical model of individual psychological resilience among nursing students. We hope that it will stimulate further research with student nurses who are the future of the nursing workforce.

## Author contributions

All authors contributed to the design of the study. BH, RO-M, CR contributed to analyses. JA, LC, DC, CR, VT, DH, CR, DGH contributed to data collection. CR took responsibility for the writing of the entire first draft. All authors contributed to revisions of the first draft.

### Conflict of interest statement

The authors declare that the research was conducted in the absence of any commercial or financial relationships that could be construed as a potential conflict of interest.
